# Navigating the Core Challenges of Atropine Therapy in Myopia: A Mechanistic and Clinical Analysis of Dose, Response, and Durability

**DOI:** 10.1155/joph/2644577

**Published:** 2026-04-21

**Authors:** Qinling Jiang, Jing Yang, Xingwei Cao, Lin Mou

**Affiliations:** ^1^ Ophthalmology Department, The Affiliated Traditional Chinese Medicine Hospital, Southwest Medical University, Luzhou, 646000, Sichuan, China, scu.edu.cn

**Keywords:** atropine, dose optimization, individualized treatment, mechanism of action, myopia control, pediatric and adolescent myopia

## Abstract

Myopia has emerged as a major global public health concern. Atropine, recognized as one of the foremost interventions for myopia control, has been validated for its efficacy and safety through a multitude of clinical studies. This article examines the most recent advancements in understanding atropine’s mechanisms of action, optimizing dosage, customizing treatment plans, and assessing its long‐term effectiveness in managing myopia. While atropine has shown impressive clinical results, there are still significant knowledge gaps surrounding its mechanisms of action, the personalization of treatments, rebound effects following cessation, and ocular side effects. Future investigations should prioritize clarifying the mechanisms of atropine, developing pertinent biomarkers for guiding individualized therapies, and investigating integrated control strategies that combine atropine with other treatments to enhance long‐lasting therapeutic benefits. As a crucial medication for the prevention and management of myopia, atropine holds significant promise for a holistic approach to myopia treatment, offering new insights and directions to tackle this public health challenge.

## 1. Introduction

Myopia has emerged as a worldwide public health issue. Myopia rates have shown an exponential rise since the 20th century, and modeling suggests that the global myopic population will be 4.758 billion by the Year 2050, making up 49.8% of the world population, especially increasing rapidly in children and adolescents [1, 2]. The accumulating body of evidence indicates that we are facing a global myopia epidemic [2, 3]. The onset and progression of myopia are influenced by both genetic susceptibility and environmental factors, including reduced outdoor time and greater time spent on near work [4–7]. Not correcting myopia can not only impose a substantial socioeconomic burden [8] but also lead to complications associated with high myopia, retinal degeneration, glaucoma, and macular degeneration, which are the major causes of irreversible vision impairment [9, 10]. The issues of myopia underscore the need for control in children and adolescents [11].

For several decades, atropine has been one of the main pharmacological interventions and the cornerstone of myopia control, particularly since the publication of the atropine for the treatment of myopia 1 (ATOM1) study [12]. Initially, high‐concentration atropine (1%, administered daily or less frequently) was used [12, 13]. More recently, much lower concentrations have been adopted, especially low‐dose atropine (e.g., 0.01% and 0.05%), which have demonstrated efficacy with fewer side effects and a lower risk of rebound [14]. However, there is still debate regarding very low concentrations, particularly 0.01%, as the low‐concentration atropine for myopia progression (LAMP) study demonstrated that this dose has minimal and clinically insignificant effect on axial length (AL) elongation compared with higher concentrations [12, 14, 15]. Despite its widespread use, the clinical application of atropine still faces several unresolved challenges. First, its mechanism of action is not fully understood and is now widely believed to be independent of accommodation suppression, contrary to early hypotheses from the late nineteenth and early twentieth centuries [16–18]. This is supported by experimental evidence, showing that atropine remains effective in species such as chickens, in which it does not induce accommodative paralysis due to the absence of parasympathetic innervation of the ciliary muscle. Current evidence indicates that topical atropine exerts a multifactorial and not yet fully elucidated effect on myopia progression, including modulation of retinal neurotransmitters, increased choroidal thickness and perfusion, reduction of scleral hypoxia, and regulation of extracellular matrix remodeling. In addition, atropine appears to act on multiple receptor pathways beyond muscarinic blockade. Overall, atropine acts through an integrated, pleiotropic mechanism affecting several pathways involved in ocular growth [16, 17, 19–24]. Second, treatment response varies considerably among individuals due to a combination of factors such as age, ethnicity, genetic background, and environmental exposure [25–28]. Third, the optimization of long‐term treatment strategies is further complicated by diminishing efficacy over time, rebound phenomena after treatment cessation, and variable adherence in pediatric patients [14, 29–31].

Given the ongoing uncertainties regarding the mechanisms of action of atropine, the significant interindividual variability in therapeutic responses, and the complexities associated with long‐term management, a timely and critical synthesis of recent evidence is warranted to enhance personalized myopia control. This article reviews the recent research on the use of atropine for myopia control with emphasis on its action mechanism, dose optimization, models for individualized treatment, and long‐term effectiveness. In the end, the importance of interdisciplinary cooperation and the merging of precision medicine are detailed.

## 2. The Biological Mechanism of Atropine Action and Its Regulation of Axial Length Growth

**FIGURE 1:**
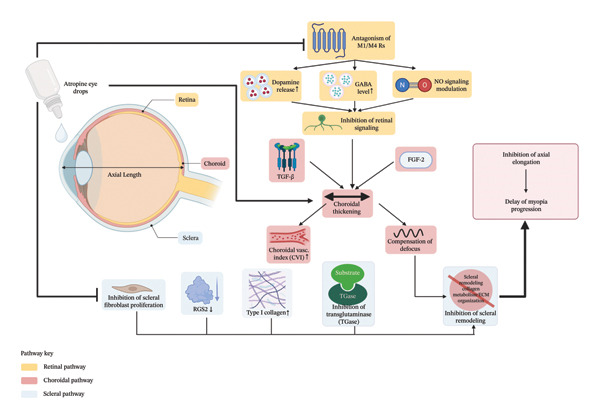
Multipathway mechanisms of atropine in myopia control. This schematic illustrates the primary biological pathways through which atropine exerts its antimyopia effects. In the retina, atropine antagonizes M1 and M4 muscarinic receptors, leading to alterations in key neurotransmitter systems, including dopamine, GABA, and nitric oxide, ultimately inhibiting retinal signaling. In the choroid, atropine induces transient thickening, enhances the choroidal vascular index (CVI), and compensates for defocus, processes that may be mediated by growth factors such as TGF‐β and FGF‐2. In the sclera, atropine directly inhibits the proliferation of scleral fibroblasts and transglutaminase (TGase) activity, while promoting the strengthening of the extracellular matrix through the upregulation of Type I collagen and the downregulation of RGS2, collectively inhibiting scleral remodeling. These coordinated actions across ocular tissues converge to inhibit axial elongation, thereby delaying the progression of myopia.

Atropine is a natural alkaloid of the Atropa belladonna plant. Atropine is a nonselective reversible mAChR antagonist that inhibits the parasympathetic nervous system by blocking the mAChR. In the past, atropine was an agent used in the eye for dilating the pupil, controlling intraocular pressure, and treating intraocular inflammation. There has been solid evidence in recent years demonstrating the effectiveness of atropine in controlling myopia, with topical application at high concentrations (e.g., ≥ 0.5%) significantly slowing AL and thereby delaying myopia [32]. We do not completely understand how atropine prevents and controls myopia on a molecular and cellular level. Recent research shows that the way atropine works to regulate eyeball growth is through different pathways and targets (Figure 1). The upcoming section presents a critical analysis of its fundamental mechanisms.

### 2.1. Beyond Accommodation Suppression: The Dominant Role of Nonaccommodative Mechanisms

The ocular accommodation reflex refers to the ability of the eye to adjust the strength of the eye’s optical system to correct for different distances and light levels. Within this part of the eye, the ciliary muscle, lens, pupil, and AL position act together in a coordinated fashion. Excessive accommodation could elongate the AL and cause or worsen the progression of myopia. Research over the years shows that atropine inhibits ciliary muscle contraction through its anticholinergic effects, relaxing accommodation and thus slowing myopia progression [12, 33]. Later studies on animal models proved that the experimental myopia still occurred even after optic nerve lesions or Edinger–Westphal (E–W) nucleus lesions. It confirms that the accommodative mechanism is not the only pathway responsible for eyeball growth regulation [16, 21]. In a study involving form‐deprivation myopia (FDM) chick models, it was found that atropine was effective in preventing the progression of myopia. The ciliary muscles of these chick models are striated, innervated by nicotinic rather than muscarinic receptors. This shows that atropine is effective not just through accommodation [22]. Subsequently, a shift in myopia research has occurred away from anterior segment accommodative mechanisms and toward posterior segment structures, particularly the regulatory roles of the retina and sclera in myopia.

### 2.2. Regulation of Retinal Signaling Pathways

The main tissue for detecting and processing visual stimuli is the retina. An increase in the AL unit is closely linked to myopia, and the retina helps regulate eyeball shape. An image blur or defocus on the retina activates intrinsic neuronal circuits. As a result, cells such as amacrine and bipolar cells release neurotransmitters. These signals cause expansion of the eye wall by affecting the choroids and sclera, which ultimately leads to axial lengthening and myopia development [34, 35]. mAChRs (M1–M5) are found in ocular tissues of the retina, choroid, and sclera. They participate in multifaceted signaling pathways in the retina. M1 and M4 receptors are mainly expressed in amacrine cells and bipolar cells of the retina. These receptors are associated with image perception and the pathogenesis of myopia [36, 37]. Atropine is thought to block these retinal receptors and lessen feedback signals, and thus inhibit ocular wall growth.

Many studies indicate that atropine works by changing how neurotransmitters function. By inducing diffuse inhibition, the intravitreal injection of atropine has been shown to enhance the release of dopamine (DA) in both intra‐ and extraocular regions. This consequently inhibits myopic development by impeding retinal processing of signals [38].

DA is an important retinal neurotransmitter that plays an important role in ocular growth, visual signal transmission, and myopia development and progression [39, 40]. Research indicates that enhancing the production and release of internal DA can effectively reduce FDM in mice [41]. Different subtypes of DA receptor also exert distinct effects on myopia progression [42]. For instance, D1 receptor agonists (e.g., apomorphine) can inhibit the progression of FDM [43]. Furthermore, antagonizing D2 receptors (e.g., by sulpiride) also protects guinea pig models of FDM, which suggests this mechanism may consist of regulating DA in the retina to stabilize neurotransmitter levels [44]. In tree shrew models, however, activation of D2 and D4 receptors has also been shown to exert antimyopia effects, underscoring the complexity of the dopamine pathway [45].

Gamma‐aminobutyric acid (GABA) is a major inhibitory neurotransmitter and essential regulator of ocular growth [46]. It was found that in rat retinal neurons, acetylcholine suppresses GABA spillover due to its muscarinic agonist actions, effect which was concentration‐dependent and was reversed by atropine which raised extracellular GABA concentrations [47, 48]. Research has shown that in lens‐induced myopia (LIM) mice, atropine downregulates the expression of GABA transporter 1 (GAT‐1), which establishes a normal excitatory/inhibitory neurotransmitter balance, which prevents the development of myopia [49]. Besides these pathways, atropine might exert antimyopia effects through the α2A‐adrenergic receptors [50] and the nitric oxide signaling pathway [51].

### 2.3. Impact on Scleral Remodeling

The outer layer of the eyeball is called the sclera. It helps protect the eyeball and helps it maintain its shape. Any change in the scleral ECM composition, scleral thinning, and reduced elasticity of the scleral ECM forms the structural basis of pathological AL elongation [23]. Atropine may slow myopia by modulating the changes that inhibit scleral remodeling. Research shows that atropine can reduce the level of transglutaminase (TGase) protein and prevent the proliferation of scleral fibroblasts (SFs) [52, 53], increase the thickness of the sclera in chick model of FDM [54] and mouse model [55], and reduce the level of scleral ECM by inhibiting DNA and glycosaminoglycan synthesis in scleral chondrocytes in chick eyes with FDM [56]. In addition, real‐time PCR analysis and atropine treatment of LIM model mice showed upregulation of the mRNA expression levels of M1, M3, and M4 receptors in the sclera [57]. Atropine‐treated FDM model mice showed reduced scleral mRNA and protein level of regulator of G‐protein signaling 2 (RGS2) but upregulated Type I collagen, which inhibited scleral remodeling and slowed down myopia progression via *in vitro* studies [58].

### 2.4. Mediating Role of the Choroid and Retinal Pigment Epithelium (RPE)

Located between the retina and sclera, the choroid chiefly provides nutrients and oxygen to the retina. Furthermore, it maintains the shape of the eyeball. The thickness of the choroid changes in association with retinal defocus, which produces a transient compensation against the refractive error induced by negative lenses [59]. Research demonstrates that choroidal thickening in LIM chicks is transiently induced by atropine [24]. Additionally, in human eyes, atropine mitigates hyperopic defocus‐induced choroidal thinning without modifying baseline thickness [60]. The net result suggests a possible choroidal effect from atropine on axial elongation. In FDM mice, atropine has been shown to restore choroidal thickness and enhance the choroidal vascular index (CVI), thereby improving choroidal microcirculation [55].

The RPE, a single layer of pigment cells located between the neural retina and the vascular choroid, is a specialized epithelium that can serve as an ideal channel for retinal growth regulatory signals to the sclera and choroid [61, 62]. The regulation of ocular growth is facilitated by the moieties of growth factors secreted by both RPE and choroid. They secrete growth factors like transforming growth factor‐β (TGF‐β), fibroblast growth factor‐2 (FGF‐2), and insulin‐like growth factor 2 (IGF‐2). As indicated by the evidence, it is believed that the choroid–scleral remodeling process may be modified by the involvement of TGF‐β [63] and activation of FGF‐2 [53] by atropine.

## 3. Mechanisms of Atropine Dosage and Strategies for Side Effect Regulation

**FIGURE 2:**
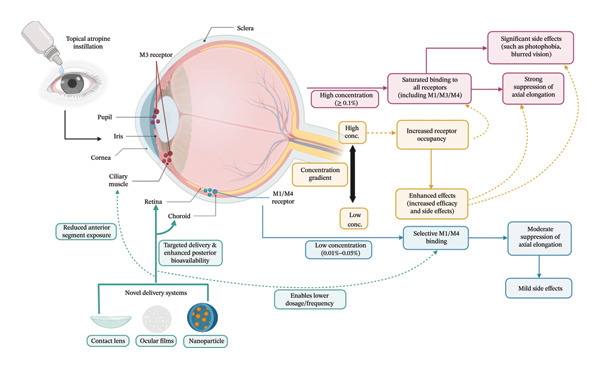
Mechanisms and dose–response of atropine therapy for myopia control. Topical atropine instillation exhibits uneven distribution in ocular tissues, resulting in concentration‐dependent effects. The low‐concentration pathway (indicated by blue arrows) involves selective binding to M1 and M4 receptors in the retina and choroid, leading to moderate suppression of axial elongation with mild side effects. Conversely, the high‐concentration pathway (depicted by red arrows) involves saturated binding to all receptor subtypes, including M3 receptors in the ciliary muscle and iris, which produces strong myopia suppression accompanied by significant side effects such as photophobia and blurred vision. The core mechanism (illustrated in the gold box) demonstrates that increased receptor occupancy along the concentration gradient enhances both therapeutic efficacy and side effects. Innovative delivery systems (represented in teal/green, such as contact lenses and ocular films) facilitate targeted delivery to the posterior segment, improving bioavailability and permitting lower dosage and frequency, thereby reducing exposure to the anterior segment. M refers to muscarinic acetylcholine receptors.

Myopia control by atropine occurs through nonselective antagonism of mAChRs with a complex concentration‐dependent profile. The multitarget mechanisms of action and the related dose–response relationship are outlined in Figure 2 of this article. The pharmacological rationale of concentration dependency, side effect management strategies, and strategies for developing new delivery systems follow in detail.

### 3.1. Analysis of the Pharmacological Mechanisms and Side Effects of Atropine at Different Concentrations

A study on the pharmacokinetics was conducted on the eyes of rabbits using a topical 1% atropine application [64]. The results of that study show that there is a dynamic distribution of the topical 1% atropine inside ocular tissues. Concentration would show a gradient from anterior to posterior, which would be the highest in the conjunctiva and the lowest in the lens after 5 h of instillation. After 24 h, atropine showed a preferential binding to posterior ocular tissues (sclera, choroid), thereby reversing the original gradient. The original gradient was that of the highest concentration in the posterior scleral (also Bruch’s) and the lowest in the conjunctiva. The concentrations of drugs in multiple ocular tissues were two orders of magnitude above their receptor binding affinity, even after 3 days. Thus, the drug may still be within the therapeutic window for retina [64]. This study revealed the characteristics of the distribution and retention of atropine in ocular tissues, which indicates the pharmacokinetics of the concentration and duration effect.

Atropine exerts its myopia control effect through nonselective antagonism of mAChRs (M1–M5).

Research has demonstrated that the effects of it depend on concentration: As concentration increases, receptor occupancy increases, creating layered pharmacological effects [65, 66]. Atropine is an amphiphilic alkaloid (log *p* = 1.83, pKa = 9.7) and must penetrate the lipophilic corneal epithelium and hydrophilic stroma to reach the eye. A part of the drug diffuses into the ciliary body, while the other part is cleared through the aqueous humor after entering the anterior chamber. The continual blood–aqueous barrier from the ciliary muscle’s capillary endothelium greatly restricts the access of drugs to muscle tissue directly [67, 68].

Studies done in a test tube have proven that ciliary muscle accommodation occurs mainly through M3 receptors. These show a display of a threshold effect on signal transduction [69]. To inhibit ciliary muscle contraction, high receptor occupancy (> 50%) is needed [70]. The low concentration of atropine targets retina/choroid M1/M4 receptors that are involved in growth control and achieve a slight suppression of AL elongation. At such low concentrations, receptor occupancy of the ciliary muscle and pupillary sphincter is minimal, resulting in milder side effects such as cycloplegia and mydriasis. In contrast, high‐concentration atropine can bind to the various receptor subtypes indiscriminately. This competes with AL inhibition significantly. However, the side effects are also rather severe, such as photophobia and near‐vision troubles. This is due to the complete blockade of M3 receptors. A meta‐analysis showed a nonlinear dose response of pupil dilation in response to atropine and reduced amplitude of accommodation. Above 0.1%, the side effects were markedly increased [71]. Clinical trial results from the ATOM2 study confirmed that 0.01% and 0.1% atropines are equally efficacious for myopia control, with the lower concentration delivering a better safety profile [14]. This relationship between concentration and effect has individualized atropine dosing.

### 3.2. Balancing and Adjustment Strategies for Dosage and Side Effects

The use of atropine in clinics depends on finding a suitable therapeutic window between the effectiveness and side effects of atropine for children and adolescents on long‐term therapy.

Numerous studies have assessed how altering the frequency of high‐concentration atropine affects efficacy and safety. In a certain animal study, a dosage of 1% atropine given every 3 days was found to have a similar efficacy in slowing myopia progression as once‐a‐day dosing. This study was done in a LIM guinea pig model. If we reduce the frequency of high‐dose atropine, it may have a similar efficacy and fewer side effects in children [72]. Zhu and colleagues examined the effectiveness and safety of 1% atropine for the long‐term treatment of myopia in Chinese children [73]. The study enrolled myopic children aged 6–12 years, having spherical equivalent (SE) refraction (SER) −2.0D to −8.0D and astigmatism ≤ 1.0D. In total, 660 children were involved. The enrolled subjects were randomly assigned to two groups. The intervention group was given 1% atropine eye drops according to a phased protocol of treatment once nightly per month for a 24‐month period (Phase I), once in two months for a 12‐month period (Phase II), and complete withdrawal for a 12‐month period (Phase III). The control group (*n* = 330) received saline drops with standard optical correction. In a two‐year interval, the 1% atropine group experienced comparable efficacy as daily dosing (around 78% and 70%) with reductions of 76.8% in myopia progression and 69.1% in axial elongation. The SE and AL measurements at the 4‐year follow‐up were significantly better in the atropine group (SE: −4.96 ± 1.22 D vs −7.28 ± 1.26 D, *p* < 0.001; AL: 25.48 ± 0.29 mm vs 26.59 ± 0.20 mm, *p* < 0.01). In addition, Phase III showed no rebound in myopia, which indicates that the low‐frequency dosing strategy is both efficacious and safe [73].

Other research has explored combination therapy and individualized dosing strategies. Some researchers have experimented with combination therapies and individualized dosing. A research study by atropine for the children and adolescent myopia progression (ACAMP) reported that compared to 0.1% atropine monotherapy, using a sequential approach with 1% and 0.01% atropine provides superior control of refractive progression and axial elongation in children with low‐to‐moderate myopia [25]. Polling et al. [74] proposed the regimen of dose adjustment based on the response. They started with a 0.5% atropine. After 1 year, the dose was increased to 1% for nonresponders, whereas the good responders gradually reduced the dose to 0.25% or even up to 0.01%. The findings suggested that the step‐down group further sustained effective myopia control without rebound, lending practical support for personalized step‐down therapies.

### 3.3. Optimizing Dosage Safety Through New Delivery Systems

To overcome the challenges of low bioavailability, frequent dosing, and high side effects resulting from traditional eye drops, different innovative ocular drug delivery systems have been devised to improve the efficacy and safety of atropine.

#### 3.3.1. Ocular Film: Prolonged Retention and Enhanced Bioavailability

Ocular films are drug delivery systems that are applied to the eyes to give medicines. Ji et al. [75] developed an atropine‐loaded ocular film (ATR film) for myopia treatment. The ATR film forms a viscous colloid liquid after application to the eye, which prolongs the residence time of the drug. Drug penetration in the conjunctiva, cornea, retina, and sclera was increased by 3.21, 2.87, 1.35, and 2.05 times, respectively. There was a pronounced improvement in the bioavailability of atropine in the sclera and retina regions along with prolonged action. The simultaneous release of a low dose of the drug in the ATR film may reduce the side effects of atropine and improve patient tolerance.

#### 3.3.2. Drug‐Loaded Contact Lenses (CLs): Combining Vision Correction and Drug Sustained Release

The drug‐loaded CLs are innovative ocular drug delivery systems, where the drug is incorporated into the CL material, allowing for prolonged drug release while being worn on the ocular surface. Fu et al. [76] prepared atropine‐responsive release CLs using an implant method for atropine loading, combined with UV‐free radical polymerization.

According to their study, atropine‐loaded CLs provided effective visual correction and therapeutic benefits, delaying the progression in the FDM guinea pig model. SEs in the 0.01% atropine eye drop group, atropine CLs‐1 group, and FDM group were 4.38 ± 0.41 D/14 days, 3.91 ± 0.36 D/14 days, and 5.47 ± 0.36 D/14 days, respectively. ALs were 8.525 ± 0.079 mm, 8.425 ± 0.071 mm, and 8.69 ± 0.083 mm, respectively. Additionally, atropine CLs‐1 demonstrated good biocompatibility. Furthermore, there was no significant irritation or toxicity to the eye in the short term [76]. Wang and others [77] reported the creation of molecularly imprinted soft contact lenses (SCLs) that can release atropine continuously for 72 h. Through the enhancement of drug bioavailability, dosing reduction and improved compliance of patients are achieved. Azi et al. [78] were the first to develop a microemulsion technology‐based atropine‐releasing CL that provides continuous drug release and fewer side effects. The toxic effect of this system on corneal cells needs to be studied further.

#### 3.3.3. Other Novel Atropine Delivery Systems

Li and his colleagues [79] created eye drops using SPSR called ATS@SPSR suspension. This system aids in continuous drug release, improves ocular bioavailability, and reduces dosing frequency through drug‐loading and sustained‐release properties of ion–exchange resins and may be a safe, effective, and convenient treatment option for pediatric and adolescent patients.

Even though new delivery systems show great promise, there are still challenges concerning high prices, validation of long‐term efficacy, and large‐scale production. Nonetheless, these findings are a noteworthy direction for future personalized and precise therapy of myopia.

## 4. Formulation of Individualized Treatment Plans

The effectiveness of atropine in controlling myopia shows considerable variation among different individuals, which depends on age, sex, genetic profile, and environmental factors.

The individualized treatment involves the formulation of specific therapeutic strategies based on the differences in patients’ genetic makeup, clinical phenotypes, behavioral habits, and environmental factors. Atropine individualized therapy, when compared with standardized regimens, can reduce adverse effects and enhance the safety, comfort, and compliance of treatment through systematic monitoring and dynamic adjustments while effectively controlling myopia development. This method combines medical monitoring and dose optimization together with behavioral interventions.

### 4.1. Impact of Individual Differences in the Efficacy of Atropine for Myopia Control

Research has shown that factors such as age, sex, myopia severity, and ethnicity can affect myopia progression [80–83]. Atropine therapy response may also be affected by these factors. The ACAMP research [25] revealed that older people showed progression with less risk of myopia. Each additional year of age was associated with a 15% lower risk of progression. Moreover, male patients demonstrated a 27% decreased risk of progression as compared to female patients. However, reasons why younger age and female gender are risk factors for myopia rebound remain unclear. Research shows that age is an important factor for atropine efficacy. A study focused on the dose adjustment of atropine for progressive myopia among European children indicated that children older than 10 years at baseline showed better and more stable treatment effects [74]. Besides, side effects of atropine are age‐dependent. According to Fu et al. [26], the use of 0.01% and 0.02% atropine in older children resulted in a greater decrease in accommodation amplitude and increased pupil dilation following administration of the eye drops. Gender also influences myopia progression. One study has shown that the ocular parameters (especially spherical power) of adolescent females change with serum estradiol throughout the menstrual cycle, indicating that the gender factor should be considered and dynamic follow‐up should be done during treatment [84].

Ethnicity is another factor influencing atropine efficacy. Studies show that the therapeutic effect of atropine varies with ethnicity and iris color. This difference may be due to a variation in the melanin content of the iris, which differs between different ethnic groups [27]. It has been confirmed *in vitro* and in animals that atropine binds to melanin [85, 86]. Further studies adopting high‐performance liquid chromatography analysis showed that the melanin content of dark irises was significantly higher than that of light irises (*p* < 0.0001) [87]. This drug and melanin binding may reduce bioavailability of physical atropine in the eyes of dark iris patients, accounting for pharmacokinetics explanation of ethnic differences in efficacy. The results indicate that individualized dosing in the future should include dose adjustment based on iris color.

### 4.2. Genetic and Environmental Factors Can Impact How Effective Atropine Is at Controlling Myopia

Myopia could be caused by genetic factors, while childhood myopia is particularly dependent on an individual’s genetic background [88]. A family history of myopia is a significant risk factor for myopia development in offspring [28]. If either or both parents are myopic, the likelihood of the child becoming myopic increases significantly. Multiple genes involved in myopia have been identified that may modulate the response to atropine via regulating mechanisms of eyeball growth and scleral remodeling [89, 90].

Xia et al. [91] explored the genetic basis for variation in efficacy of orthokeratology (Ortho‐K) lenses in children through whole‐genome sequencing and analysis of genetic variation. Moreover, it is suggested that genetic background may regulate individual differences in treatment response. In a similar fashion, Hsiao et al. [92] employed bioinformatics strategies. Subsequently, they found that atropine could hamper AL growth. This is accomplished by regulating the expression of genes involved in the cell cycle, proliferation, differentiation, and extracellular matrix remodeling. These include FOXP3, CDT1, and PLXDC1. They also include miRNAs such as hsa‐miR‐2682‐5p [92]. A retrospective study suggested that the genetic background of myopia could modify the efficacy of atropine, as 0.01% atropine was reported to be more effective in controlling myopia in the case of children born through normal delivery with a mother without a myopic history [28]. According to Tkatchenko et al. [93], there is a link between genomic studies and pathways of myopia development. The efficacy of antimyopia drugs (e.g., atropine) may be regulated genetically. They describe that genomics and pharmacogenomics will be instrumental in the future development of individualized myopia drugs [93].

In addition, interactions between genes and the environment are very important for myopia development. Some environmental risk factors influence the ability of genes to cause myopia in children [94]. Near‐work visual load due to modern education patterns and less time spent outdoors are two primary risk factors [95]. As per a study performed to evaluate the efficacy of low‐concentration (0.01%) atropine for myopia control and drug efficacy in an Indian population, children with more than 2 h of outdoor activity showed better efficacy [96]. Also, the COVID‐19 gathering restrictions have necessitated children to resume learning mostly in the home, increased their near‐work time, and lessened outdoor time. This altered the effectiveness of atropine treatment during the pandemic [97]. Erdinest et al. [98] in Israeli children treated with 0.01% atropine found similar results.

### 4.3. Integration of Individualized Treatment and Precision Medicine

To translate individual differences into clinical settings, we must depend on the precision medicine’s multimodal assessment framework.

The precision medicine concept refers to a “patient‐centric” development of a treatment plan based on a comprehensive analysis of the patient’s biological characteristics. Prior to the beginning of treatment, a complete baseline assessment, such as SE, AL, biometry, visual load, family history, lifestyle, and educational level, must be done. Throughout treatment, high‐resolution imaging, such as monitoring choroidal thickness, changes through optical coherence tomography (OCT), and precisely assessing rates of axial elongation through ultrasonic biometry, provides an objective basis for evaluating treatment response and dose adjustment [99, 100]. Rapid advancements in genomics have created an opportunity to identify high‐risk groups for myopia [88, 101]. Studies suggest that the effectiveness of antimyopic medications, including atropine, may vary according to individual genetic differences. It is noted that pharmacogenomic screening in the future would facilitate predictions of pharmacokinetics and adverse reaction risks toward the development of individualized antimyopia treatment [93]. Additionally, the use of artificial intelligence (AI) and machine learning enables the application of big data. Ophthalmic imaging, biomarkers, and electronic medical record data have been blended together to commence developing efficacy prediction models. They can assist clinicians in risk stratification, predicting treatment response, and assessing long‐term efficacy, thus enhancing decision‐making accuracy [102, 103].

To sum up, personalized atropine treatment holds promise for precision medicine in myopia control. When preparing a personalized treatment plan, it must consider the patient’s age, gender, ocular structure, genetic background, and environment. Treating a patient this way would improve the efficacy and long‐term safety of treatment. With the development of larger multicenter studies and application of AI technology, treating myopia will move toward personalization. This will enable a more “data‐driven” than “experience‐driven” approach.

## 5. Clinical and Long‐Term Efficacy Studies

For a clear outline of all the clinical studies discussed in this chapter, the core design, population, interventions, and main findings of these have been summarized in Table 1 (see Table 1).

**TABLE 1:** Summary of key clinical trials on atropine for myopia control.

Study (reference)	Design	Participants (age, SER)	Intervention groups	Duration	Primary outcomes and key findings
Comparison of the effect of atropine and cyclopentolate on myopia (May‐Yung et al. [104])	RCT (NR)	96 children, 6–14 years, −4.00 D to −0.50 D	1% atropine; 1% cyclopentolate; placebo (saline)	12 mo	Both atropine and cyclopentolate slowed myopia progression, with atropine being more effective.

ATOM1 study (Wei‐Han Chua et al., [12])	RCT, DB	400 children, 6–12 years, −6.00 D to −1.00 D	1% atropine; placebo (saline)	24 mo	SER progression: 0.28 (1% atropine) vs 1.20 D/year (placebo). 80% reduction in myopia progression in the atropine group compared to placebo. Significant side effects noted.

ATOM1 study (washout period) (Louis et al. [31])	RCT, DB	Cohort from ATOM1, 6–12 years	Washout of 1% atropine; washout of placebo	12 mo	Significant rebound effect after cessation of 1% atropine. Induced side effects were partially reversible upon discontinuation.

ATOM2 study (Phase 1) (Audrey et al. [14])	RCT, DB	400 children, 6–12 years, SER ≤ −2.00 D	0.5% atropine; 0.1% atropine; 0.01% atropine	24 mo	Efficacy was dose‐dependent. The 0.01% concentration offered the best safety profile.

ATOM2 study (Phase 2—Washout) (Audrey et al. [105])	RCT, DB	Cohort from ATOM2, 6–12 years	Washout of 0.5%, 0.1%, and 0.01% atropine	12 mo	Rebound magnitude after cessation was dose‐dependent. The 0.01% group demonstrated the best overall efficacy over 3 years due to minimal rebound.

LAMP1 study (Phase 1) (Jason et al. [15])	RCT, DB, PC	438 children, 4–12 years, SER ≤ −1.00 D	0.05% atropine; 0.025% atropine; 0.01% atropine; placebo (saline)	12 mo	0.05% atropine was the most effective concentration, reducing myopia progression by 67% (SER).

LAMP2 study (Phase 2) (Jason et al. [29])	RCT, DB	438 children, 4–12 years, SER ≤ −1.00 D	0.05%, 0.025%, and 0.01% atropine (continued); Placebo ⟶ 0.05% atropine (switch)	24 mo (continued group) 12 mo (placebo‐to‐atropine switch group)	0.05% atropine maintained the best efficacy over the 2‐year treatment period.

LAMP2 study (Phase 3) (Jason et al. [106])	RCT, DB	438 children, 4–12 years, SER ≤ −1.00 D	Three study arms are as follows: 1. Continued‐treatment groups (0.05%, 0.025%, 0.01% atropine) 2. Washout groups (cessation of 0.05%, 0.025%, 0.01% atropine) 3. Placebo‐to‐atropine switch group (to 0.05% atropine)	Continued and switch: 36 mo total Washout: 12 mo (Year 3)	Rebound after cessation, milder with lower concentrations and older age; continued treatment superior to washout; 0.05% atropine provided the highest net benefit over 3 years

LAMP2 study (prevention) (Jason et al. [107])	RCT, DB, PC	474 children, 4–9 years, 0.00 D to +1.00 D (nonmyopic)	0.05% atropine; 0.01% atropine; placebo (saline)	24 mo	0.05% atropine significantly reduced the incidence of myopia compared to placebo.

CHAMP study (Karla et al. [108])	RCT, DB, PC	489 children, 6–10 years, −6.00 D to −0.50 D (mITT)	0.01% atropine (PF); 0.02% atropine (PF); placebo (vehicle)	36 mo	−1.04 D (0.01%) vs −1.28 D (placebo). 0.01% effective; 0.02% not superior. Both concentrations safe.

Low‐Dose 0.01% Atropine Eye Drops vs Placebo for Myopia Control: A Randomized Clinical Trial (Michael et al. [109])	RCT, DB, PC	187 children, 5–12 years, −6.00 D to −1.00 D (US)	0.01% atropine; placebo (vehicle)	24 mo	No statistically significant difference in SER progression between groups.

Myopia Control in Caucasian Children with 0.01% Atropine Eye Drops: 1‐Year Follow‐Up Study (Dovile et al. [110])	Non‐RCT, NDB	121 children, 6–12 years, −5.00 D to −0.50 D (Caucasian)	0.01% atropine; untreated control	12 mo	No statistically significant difference vs control. A slowing trend observed in treatment group.

Myopia Outcome Study of Atropine in Children (MOSAIC Study) (James Loughman et al., [111])	RCT, DB, PC	250 children, 6–16 years, SER ≤ −0.50 D (primarily white)	0.01% atropine; placebo (vehicle)	24 mo	Significant reduction in AL elongation (−0.07 mm); no significant difference in SER. Efficacy influenced by race/iris color.

*Note:* mo: months.

Abbreviations: AL, axial length; DB, double‐blind; mITT, modified intention‐to‐treat; NDB, non–double‐blind; NR, not reported; PC, placebo‐controlled; PF, preservative‐free; RCT, randomized controlled trial; SER, spherical equivalent refraction.

In the LAMP2 (Phase 3) trial, the “continued‐treatment” groups received the same atropine concentration throughout, the “washout” groups ceased treatment after 24 months, and the “switch” group consisted of participants originally assigned to placebo who were switched to 0.05% atropine at 24 months. Outcome data are presented as mean values or key comparative results.

### 5.1. Existing Large‐Scale Multicenter Clinical Trials and Efficacy Evaluation

Atropine is most recognized for its myopia treatment and control. Its clinical effectiveness has been confirmed with large‐scale and highly rigorous multicenter randomized trials such as the ATOM, LAMP, and childhood atropine for myopia progression (CHAMP) studies.

The earliest randomized controlled trial, with topical atropine to control myopia progression, showed that 1% atropine was more effective than 1% cyclopentolate or placebo. Nonetheless, since AL measurements were not made then, this did not allow for the assessment of the effect of atropine on AL elongation [104]. The ATOM1 study, released in 2006, proved the effectiveness of 1% atropine quite soundly. This trial lasted 2 years and was prospective randomized trail with a double‐blind sample. It had 400 children as subjects, and they aged 6–12 years. Moreover, it had SEs ranging from −1.00 D to −6.00 D with astigmatism ≤ 1.50 D. The children were randomly assigned to either a 1% atropine or a placebo group. After 2 years, the myopia progression was much lower in the atropine group than the placebo group (0.28 ± 0.92 D vs. 1.20 ± 0.69 D). AL elongation was also much lower than that of the control group (0.02 ± 0.35 mm vs. 0.38 ± 0.38 mm). According to this finding, 1% atropine significantly slowed the progression of myopia and AL elongation in Asian children with moderate‐to‐low myopia [12]. The side effects associated with 1% atropine, namely, photophobia and near‐vision blur, restrict its use in children and adversely affect compliance. ATOM2 aimed to determine whether lower doses of atropine could maintain efficacy while reducing side effects. The study compared the efficacy and safety of 0.5%, 0.1%, and 0.01% atropine. The study assigned 40 children randomly into three groups in the ratio of 2:2:1. After 2 years of the treatment, the myopia progression was −0.30 ± 0.60 D in the 0.5% group, −0.38 ± 0.60 D in the 0.1% group, and −0.49 ± 0.63 D in the 0.01% group. The corresponding AL elongation was 0.27 ± 0.25 mm, 0.28 ± 0.28 mm, and 0.41 ± 0.32 mm. The group that used 0.01% had the mildest side effects among the groups though differences in efficacy were small. According to their findings, 0.01% atropine was effective but safer. Therefore, this drug should be regarded as a clinically relevant agent for myopia control [14].

This double‐blind, placebo‐controlled randomized clinical trial of LAMP study provides Level 1 evidence for low‐concentration atropine for the first time. In the LAMP study’s first phase (LAMP1), it was the 0.05% atropine group which demonstrated the best efficacy in controlling SE progression and AL elongation [15]. In the fourth group, the average SE changes over a year were −0.27 ± 0.61 D, −0.46 ± 0.45 D, −0.59 ± 0.61 D, and −0.81 ± 0.53 D (*p* < 0.001), respectively. AL elongation was 0.20 ± 0.25 mm, 0.29 ± 0.20 mm, 0.36 ± 0.29 mm, and 0.41 ± 0.22 mm, respectively (*p* < 0.001) [15]. According to the overall efficacy and side effects conclusion by the researchers, the best concentration was 0.05% atropine [15]. The study LAMP2, which is the second phase, established the long‐term efficacy and safety of different atropine concentrations at a 2‐year follow‐up. After 2 years of treatment, SE progression in the 0.05%, 0.025%, and 0.01% groups was 0.55 ± 0.86 D, 0.85 ± 0.73 D, and 1.12 ± 0.85 D, respectively, and AL elongation was 0.39 ± 0.35 mm, 0.50 ± 0.33 mm, and 0.59 ± 0.38 mm, respectively. The 0.05% atropine was twice as effective as the 0.01%. After the placebo group in LAMP1 switched to 0.05% atropine, the second‐year myopia progression slowed significantly (SE: second year 0.18 D vs. first year 0.82 D, *p* < 0.001; AL: second year 0.15 mm vs. first year 0.43 mm, *p* < 0.001), further supporting the superiority of the 0.05% [29]. At the same time, the LAMP2 study looked at the effect of low‐concentration atropine on delaying myopia onset. The study recruited 474 nonmyopic children aged 4–9 years who were randomly allocated to 0.05% atropine (*n* = 160), 0.01% atropine (*n* = 159), and placebo (*n* = 155). After 2 years, myopia incidence rates were 28.4% (33/116), 45.9% (56/122), and 53.0% (61/115) for the three groups. The occurrence rates of rapid myopia progression were 25.0%, 45.1%, and 53.9%. The outcomes showed that only 0.05% atropine was significantly effective in preventing the myopia onset as well as rapid progression. Moreover, this formulation was statistically not different from placebo for 0.01% atropine [107]. Studies in the LAMP series established that 0.05% atropine is superior for myopia control and provided safety data on long‐term treatment with low‐concentration atropine.

The CHAMP study is a 3‐year‐long, multicenter clinical trial to see how effective of 0.01% and 0.02% atropine in preventing the progression of myopia in children. Use of 0.01% atropine, in comparison with placebo, exhibited superiority in increasing treatment response rate (OR = 4.54, 95% CI 1.15–17.97, *p* = 0.03). Furthermore, atropine significantly reduced the progression of myopia (−1.04 D vs. −1.28 D, *p* < 0.001) and also AL elongation (0.68 vs. 0.81 mm, *p* < 0.001). The response rate in the 0.02% atropine group did not significantly improve, and its overall efficacy was less than that in the 0.01% group. According to the study, 0.01% atropine should be considered as an option to intervene and slow myopia progression [108].

Most importantly, responses to atropine therapy vary by population. According to a study involving children in the United States, children administered with 0.01% atropine did not show signs of significantly slower SE progression or AL elongation compared to placebo [109]. Likewise, another research conducted on the use of atropine for myopia control in Caucasian children reported similar findings [110]. The MOSAIC study is an investigator‐initiated, double‐blind, randomized controlled trial that enrolled myopic children aged 6–16 years. 2:1 randomization to 0.01% atropine vs control was performed [111]. After 2 years, SE did not differ significantly between the atropine party and placebo party (effect size = 0.10 D and p = 0.07). The atropine group showed markedly diminished AL elongation (difference = −0.07 mm, *p* = 0.007) [111]. Analysis of specific groups indicated significant changes to the SE and AL of white children, who exhibited a corrected final SE change of 0.14 D (*p* = 0.049). Additionally, they had a corrected final AL elongation of −0.11 mm (*p* = 0.002). The results indicate that a potential relationship exists between race, iris color, and varying effectiveness of atropine treatment [111]. Moreover, according to an analysis of LAMP data, age is an important modulator of treatment response. Younger children generally respond less well to lower concentrations (0.01% and 0.025%) than older children, and so require higher concentrations (0.05%) to achieve similar control effects [112].

In conclusion, large‐scale clinical trials show that atropine is effective. At the same time, factors like concentration, race, and age significantly modulate and impact its effectiveness. Another important consideration is whether those effects can be sustained over the long haul, and whether any side effects can be managed.

### 5.2. Long‐Term Efficacy and Side Effect Monitoring

Atropine has proven effective for short‐term myopia control, but its long‐term efficacy, post‐treatment response, and potential cumulative side effects continue to be a major area of concern in clinical practice and research.

Treatment duration extension is associated with two major challenges: the attenuation of response and the rebound effect after drug cessation. A former is the way in which efficacy declines over time, while the latter is the acceleration of myopia after stopping the treatment. Li et al. [30], in 10‐ to 20‐year follow‐ups of children from the ATOM1 (1999–2003) and ATOM2 (2006–2012) studies, evaluated the long‐term efficacy and safety of various concentrations of atropine (0.01% to 1%). It was observed that although early treatment with atropine for 2–4 years could delay myopia progression, in adulthood, there was no statistically significant difference in SE or AL between the treatment group and the control group. This finding shows short‐term effect limits, suggesting to maximize treatment benefit, either continuous treatment or better stopping strategies should be considered.

Cessation management is another pivotal issue. The severity of the rebound effect after stoppage may be closely related to the concentration of the drug, total duration of treatment, age at stoppage, and baseline severity of myopia. According to the evidence, rebound is greater after the cessation of higher concentrations of atropine. For example, the ATOM1 study [31] reported that at 1 year after 1% atropine was stopped, SE progression in atropine‐treated eyes became −1.14 ± 0.80 D/year, which was significantly greater than that in the placebo group (−0.38 ± 0.39 D/year, *p* < 0.0001). In the third phase of the LAMP trial, study findings [106] demonstrated that stopping treatment with 0.05%, 0.025%, or 0.01% atropine led to increased SE progression and AL elongation compared with ongoing treatment. Rebound effects were milder in older children and those treated at lower concentrations. Lee and colleagues [113] reported a mild myopia rebound in Australian children (SE progression: −0.41 D vs placebo group −0.28 D; AL elongation: 0.20 mm vs 0.13 mm) 1 year after ceasing 0.01% atropine use. However, there were no significant differences in cumulative myopia progression between the groups for 3 years. Also, the abrupt discontinuation of 0.01% atropine may lead to disturbance in choroidal thickness due to physiological changes in children [99]. To lessen rebound, concentration tapering has been proposed to be an effective strategy. Nonetheless, there remains no standardized protocol to taper the use of these medications and further study is warranted.

It is important to monitor the safety of pharmacological treatments in the long term. With prolonged use, particularly at higher concentrations, side effects of atropine may become apparent. 1% of the treatment of atropine in the Yen et al. study showed universal photophobia, which was attributed to a high dropout rate [104]. The ATOM1 trial documented adverse events associated with 1% atropine, including allergic reactions (4.5%), glare (1.5%), and blurred near vision (1%) [12]. Low‐concentration atropine is generally regarded as safer than its high‐concentration counterpart, yet it may still have a risk of adverse long‐term effects on the eyes, including retinal health, photophobia, and accommodation. The follow‐up study of LAMP2 [29] was done to monitor the patient’s progress for a longer period. It revealed that even with 0.01%–0.05% atropine, 30% of patients used photochromic lenses to reduce their photophobia during the initial phase of treatment. Other studies have reported fluctuations of intraocular pressure with topical low‐concentration atropine, which, although stays in the normal range, still needs monitoring [114].

For that reason, there is a necessity for a robust and long‐term follow‐up and monitoring system. The visual acuity refraction AL intraocular pressure, corneal thickness accommodation fundus, and other assessments must be carried out at baseline and during each follow‐up visit, and enhanced patient education for better compliance toward treatment and greater ability to tackle symptomatic burden. It is important to keep on monitoring a patient so that the long‐term risk can be reduced and the benefits of the treatment can be maximized.

### 5.3. Studies in Pediatric and Adolescent Patients on Efficacy: the Window of Opportunity and Challenge for Development

Myopia intervention and the clinical use of atropine may be targeted at childhood and adolescence as the primary population. The special characteristic of this population is that it is not only a large one that will be affected it is estimated that by 2050 approximately 740 million children and adolescents worldwide will be affected by myopia, and their developing visual systems have specific responses to the drug, leading to clinical management issues [1].

#### 5.3.1. Age‐Related Efficacy: From the Optimal Intervention Window to Response Attenuation

Atropine is a useful agent for the control of myopia in children and adolescents, as this group shows faster AL elongation and myopia progression, while early intervention delays myopia significantly [32, 115]. Atropine effectiveness is influenced by age, having a clear dependence on the age of an individual.

##### 5.3.1.1. Childhood—the Highly Effective Window for Intervention (6–12 years)

According to data from a European pediatric population aged 4–17 years, Rauscher et al. [116] found that AL elongation occurs more rapidly in younger children and slows with age. Further confirmation of this age‐dependent pattern was seen in a Chinese cohort, when a prospective study showed the deceleration of the AL elongation rate at age [117]. The evidence points toward the idea that AL growth in children depends on age. The critical window for myopia intervention likely occurs during the period of rapid AL growth between 6 and 12 years of age. Clinical trials such as ATOM2 have established that low‐concentration atropine use (0.01%) in this stage can greatly delay myopia progress (50% lower over 2 years [14]). Also, the LAMP study suggested that atropine is time‐dependent. In the 0.05% atropine group, starting treatment before age 8 had a 3‐year AL growth inhibition rate of 54% vs 32% in those starting at age 12 [106]. This is the first direct evidence of the efficacy of early intervention. This difference is probably due to heightened scleral tissue plasticity during the initial stages of development and increased cholinergic pathway sensitivity to the compound. However, this does require further research.

##### 5.3.1.2. Response Attenuation During Adolescence

The eye drop atropine is very effective in controlling myopia in children. However, the effectiveness is progressively reduced with age, especially in teenagers (> 12 years), where some patients may exhibit a response attenuation phenomenon. The CHAMP investigation revealed that the 0.01% atropine treatment for myopia suppression was only 22% in the third year in adolescents aged 13–15 years. This was much lower than the 51% in the first year [108]. The decline may be due to ocular maturation and the natural slowing of AL elongation during adolescence [118], which limits the possibility of drug intervention. The results showed that the efficacy of atropine depends on the development of the individual and the development of the eye.

#### 5.3.2. Ocular Safety During Development: Potential Impacts on Visual System Maturation

When giving tropine to children and teenagers, we should not only watch for normal side effects but also consider whether it will affect how their visual system matures.

The process of ocular accommodation is centered on the ciliary muscle, which is still functionally shaped during development. Atropine exposure in the long term may have two effects. The first could be a short‐term reversible suppression of accommodative function. After 2 weeks of using 0.01%, 0.02%, and 0.03% atropine, the children showed mean reductions in accommodation amplitude of 5.23 D, 9.28 D, and 9.32 D, respectively, with a large rebound postdiscontinuation [119]. Second, potential long‐term adaptive changes occurred. According to the ATOM2 study, ciliary muscle function only partially recovered 12 months after stopping higher dose atropine (0.1% and 0.5%). This indicates that longer treatment might affect ciliary muscle function permanently [105].

In addition, drug‐induced pupil dilation can have dual effects on visual development: On the retinal level, pupil dilation increases the incidence of short‐wavelength light that has been confirmed to tightly relate with retinal DA release [120], and the DA signaling pathway is a key mechanism regulating AL growth [43]. As a result, the dilation of pupils which atropine may cause might not have any direct connection with the regulatory system. But it may change the spectrum, and perhaps this needs to be determined in the fixed spectrum. When the pupil dilates more at the optical level, this can cause extra higher order aberrations. A study on the subject has shown that after 1% atropine‐induced mydriasis under a 6‐mm pupil diameter, the root mean square of the total higher order aberration or HOA increased from 0.333 ± 0.093 μm to 0.377 ± 0.095 μm (*p* < 0.01) [121]. It remains uncertain whether the blurred visual signals will be regarded as “abnormal visual experience” that interferes with the fine‐tuning of visual cortical circuit maturation during critical periods. According to visual development theory, there is a large gap between the basic properties of neuronal receptive fields and immature visual behavioral function during infancy [122]. Consequently, clarifying the possible impact of atropine‐induced lasting optic modifications on central neural plasticity is an important issue, which needs to be studied promptly using interdisciplinary study (e.g., neuroelectrophysiology and behavior).

To conclude, myopia progression can be effectively controlled, while also minimizing the interference of the drug with visual development and daily function is mainly concerned, which further favors the position of low‐concentration atropine as the first‐line clinical choice.

#### 5.3.3. Adherence Challenges and Multidimensional Optimization Strategies

The long‐term use of atropine for controlling myopia in children and adolescents encounters a range of issues. Chief among them is poor adherence. There are many reasons for nonadherence. They include limited self‐ability to self‐administer medicine in children. Also, side effects like photophobia, blurred near vision, and ocular irritation can affect studies and daily life. Furthermore, limited parental awareness regarding the risks of long‐term myopia progression and the importance of consistent medication remains a concern. Finally, there is lack of supportive treatment environment and access to eye care. These will affect treatment persistence and hence treatment efficacy [123]. As a result, optimizing adherence has become a major focus to enhance treatment effects. The strategies for prevention and control of hypertension are now shifting from the monodrug approach to a patient‐centered multidimensional synergy management model based on combined therapy, building lifestyle changes, and intelligent support systems to increase tolerability and sustainability strategies.

##### 5.3.3.1. Combination Therapy: Reducing the Side Effect Burden Through Mechanistic Synergy

Combination treatments show additional effects or increased efficacy when given together. They exert their effect via different, complementary mechanisms. This ensures that most of the effect could be retained when the dose of atropine is lowered. This translation of activity would subsequently lead to a reduction of atropine side effects, resulting in greater tolerability and acceptance. At present, the clinical practice has largely adopted the combination of atropine with optical interventions.

The synergy of atropine with Ortho‐K lenses is a classic case of pharmacological and optical mechanism synergy. Worn overnight, Ortho‐K lenses reshape the cornea. This process flattens the central zone. In turn, it creates a mid‐peripheral ring of myopic defocus. As a result, daytime uncorrected vision becomes clear. Also, they reduce atropine‐induced daytime photophobia and near‐vision blur. Simultaneously, atropine inhibits AL elongation at the biochemical level [124]. Together, their combination offers a synergistic effect. According to research, the possible use of lower atropine concentrations (e.g., 0.01%) in such combination regimens significantly improves patients’ subjective feelings and medication experience [125].

As optical technology improves, childhood myopia is being treated with spectacles and soft lenses with defocus incorporated multiple segment (DIMS) lenses emerging as a new combination therapy option. DIMS lens and atropine show good synergism. The AL annual increase was 0.22 ± 0.14 mm in the DIMS‐only treatment group. However, the AL change in the 0.01% atropine combined with the DIMS treatment group was 0.15 ± 0.15 mm after 12 months. This suggests highly significant additive effects, especially in older children [126].

A new avenue of research is investigating combinations with neuromodulation therapies. The combination of low‐dose atropine together with auricular acupoint stimulation has unique potential. Auricular stimulation may further enhance the cholinergic action of atropine through the activation of the vagus nerve by modulating the autonomic nervous system, playing a role in enhancing ocular blood circulation response and relieving accommodative spasm [127]. A randomized controlled trial showed that auricular acupoint stimulation combined with 0.01% atropine slowed myopia progression compared with using 0.01% atropine alone in Chinese children over 6 months (the mean SE difference between groups was 0.13 D) [128]. Despite being imperfectly understood in its exact neurophysiological mechanisms, it offers a noninvasive option for those who are intolerant to optical correction or wish for adjunctive therapy.

##### 5.3.3.2. Lifestyle Interventions: Building a Supportive Environment for Proactive Health Management

Lifestyle modification interventions seek to create a favorable, supportive environment for pharmacological treatment by modifying the behavioral and environmental risk factors. This strategy will control the progression of myopia and also improve treatment adherence by enhancing health self‐efficacy in children and adolescents.

The primary intervention in treating myopia is to include “outdoor activity” as part of the “light nutrition prescription.” Its mechanism of action includes the following: First, increased outdoor light intensity induces DA release in the retina, which inhibits AL elongation. Second bright outdoor light generates pupil constriction, increases depth of field, reduces peripheral retinal defocus, and minimizes blurred image stimuli (optical), thereby slowing myopic progression [129]. Meta‐analyses have shown that increasing outdoor time significantly slows down the progression of SE and AL, and reduces the incidence of myopia [130]. As such, clinicians should recommend at least 2 h of outdoor activity per day or 14 h per week, in which health education assists in emphasizing to parents that outdoor activity is as essential as prescription in controlling myopia [131].

In addition, visual behavior can be regulated by promoting the “20‐20‐20” rule (i.e., every 20 min of near work, looking at an object 20 feet away for 20 s) [132]; optimizing reading and writing environment, which includes proper and uniform lighting [133]; maintaining the correct sitting posture of “one foot, one fist, one inch”; and using adjustable desk and chairs [134]. Besides, limit the screen timings for entertainment each day [135].

With pharmacological treatment, these behavioral interventions provide a multidimensional protective system. This motivates families of patients and children to be transformed from passive medication executors to active health managers, making them more aware and supportive of treatment at the cognitive and behavioral level.

##### 5.3.3.3. Digital and Systematic Support: Achieving Sustainable Long‐Term Management

We should formulate comprehensive prevention and control strategies based on the essential integration of digital technology to provide a systematic guarantee for long‐term adherence. A combined emphasis on technology and system‐level support can help address adherence issues.

Chronic diseases may be managed using smart pill boxes and applications that use gaming elements. Intelligent adherence management tools support patient adherence via medication reminders, automatic recording, and feedback mechanisms that offer incentives [136]. The tools generate reports to visualize medication, while some applications that use gamified strategies, points, and badges, offer active participation motivation for pediatric patients [137]. The technology, though still in its inception in terms of myopia control, has broader applicability and efficacy that signifies great potential in helping manage atropine treatment. Hence, promoting these digital tools is a major technological pathway to developing a systematic adherence support system.

Establishment of structured patient education/peer support systems, based on technological support, is essential. Support models like these have proven beneficial in enhancing treatment beliefs and adherence in chronic disease management like diabetes and asthma [138, 139]. In the field of myopia control, other models have also shown immense potential. Research shows the regular push of structured health education content to parents via social media (e.g., WeChat) enhances family knowledge of myopia and behavior interventions, which may delay onset and progression of myopia to some degree [140]. Moreover, taking the initiative to set up online interactive parent communities led by healthcare providers will offer caregivers the forum to share experiences with one another along with answering each other’s questions and providing emotional support. As we have already noted, chronic disease management experience indicates that the peer support model fosters long‐term adherence by combating feelings of loneliness and by ensuring ongoing surveillance [141, 142]. While high‐level RCT evidence for myopia prevention communities is still developing, the general theories of social support, behavioral modeling, and peer motivation were shown to have some general legitimacy and good potential in enhancing parental confidence and adherence.

To conclude, the strategy to improve adherence to atropine treatment in children and adolescents must be multidimensional and systematic. The challenges in long‐term management of pediatric myopia can be overcome only by reducing the medication burden via combination therapy, creating a positive environment with lifestyle intervention and providing continuous support with intelligent and structured support systems for maximization of effectiveness of myopia prevention and control.

In conclusion, large‐scale clinical trials have shown that atropine use (especially at low concentration) is effective for myopia prevention and control in children and adolescents. While this advantage has been established, it will only be translated into clinical practice after successfully addressing the key challenges of individual efficacy, long‐term adherence, rebound upon cessation, and side effect management. When it is used for a long time, vigilance against possible cumulative effects must take place and systematic monitoring must be introduced. Being the major beneficiary population, the characteristics of growth and development and the persistent adherence bottlenecks of children and adolescents necessitate the constant balance of efficacy, safety, and convenience, in order to continuously explore better treatment and management schemes for this population. These difficulties also direct future research in important directions.

## 6. Discussion

According to the results, low‐concentration formulations (especially in the 0.01%–0.05% range) are a key to axial elongation and myopia progression. Nevertheless, achieving optimal long‐term effects remains challenging due to a poorly understood mechanism, significant interindividual variability, poor long‐term adherence, and rebound effects on withdrawal (discontinuation). To better deal with these challenges, future studies must clearly clarify their multitarget mechanisms of action at the molecular and cellular levels. Based on such findings, we discover biomarkers predictive of the efficacy of treatment. In addition, we need to integrate multimodal data such as genomics and imaging technologies, beyond the conventional differentiation based on phenotypes, to improve clinically useful predictive models of treatment response. At the clinical management level, it is significant to explore dynamic dosing regimens based on treatment responses (drugs administered as necessary), develop synergistic combination therapies (e.g., optical defocus), enhance adherence and convenience through novel drug delivery systems, and establish a comprehensive management system, consisting of structured patient education and long‐term follow‐up to sustain long‐term efficacy. In conclusion, despite the proven efficacy of atropine in myopia management, further understanding of the mechanism of action and development of clinical strategies will enhance its efficacy in children and adolescents. Future studies should focus on clarifying underlying mechanisms, developing individualized strategies, and optimizing long‐term management through innovative research designs. Multidisciplinary collaboration can take myopia control and prevention into the next era of increasing precision, safety, and effectiveness.

## 7. Conclusion

Atropine is considered a well‐accepted solution to use for slowing myopia progression in children. However, its application remains limited due to the more complex underlying mechanisms involved, high interindividual variability, and the issues faced with chronic use. In order to overcome these obstacles, the field will need to evolve from a “one‐size‐fits‐all” treatment to a treatment based on precision medicine. A focus on the mechanism of action will inform the personalization of strategies, like targeted dosing, new delivery technologies, or a multidrug approach, and their incorporation into an organized long‐term plan for more effective and sustainable myopia control.

## Funding

This work was supported by the Luzhou Municipal People’s Government—Southwest Medical University Science and Technology Strategic Cooperation Project (Grant No. 2024LZXNYDJ043).

## Ethics Statement

This is a review article and does not involve any original studies with human participants or animals by the authors.

## Conflicts of Interest

The authors declare no conflicts of interest.

## Data Availability

No new data were generated or analyzed in support of this review. All data and literature discussed in this manuscript are available from the corresponding author upon reasonable request or are publicly accessible through the cited references.
